# Polyethyleneimine-Assisted Fabrication of Poly(Lactic-Co-Glycolic Acid) Nanoparticles Loaded with Tamibarotene (Am80) for Meflin Expression Upregulation

**DOI:** 10.3390/jfb16100368

**Published:** 2025-10-01

**Authors:** Tomoya Inose, Tadashi Iida, Hiroki Kawashima, Atsushi Enomoto, Maki Nakamura, Ayako Oyane

**Affiliations:** 1 Research Institute of Core Technology for Materials Innovation, National Institute of Advanced Industrial Science and Technology (AIST), AIST Tsukuba Central 5, 1-1-1 Higashi, Tsukuba 305-8565, Japan; a-oyane@aist.go.jp; 2 Department of Gastroenterology and Hepatology, Graduate School of Medicine, Nagoya University, 65 Tsurumai-cho, Shouwa-ku, Nagoya 466-8550, Japan; iida.tadashi.s6@f.mail.nagoya-u.ac.jp (T.I.); kawashima.hiroki.p5@f.mail.nagoya-u.ac.jp (H.K.); 3 Department of Pathology, Graduate School of Medicine, Nagoya University, 65 Tsurumai-cho, Shouwa-ku, Nagoya 466-8550, Japan; enomoto@iar.nagoya-u.ac.jp

**Keywords:** Meflin, polyethyleneimine (PEI), poly(lactic-co-glycolic acid) (PLGA), tamibarotene (Am80), nanoparticle, cancer-associated fibroblast (CAF)

## Abstract

Tamibarotene (Am80) is a promising anti-tumor drug that induces the expression of Meflin (a glycosylphosphatidyl inositol-anchored protein) in cancer-associated fibroblasts, thereby improving the tumor microenvironment. However, Am80, which is approved only for oral administration owing to its poor water solubility, has the challenge of poor tumor penetration. In this study, we developed poly(lactic-co-glycolic acid) nanoparticles loaded with Am80 (Am80–PLGA nanoparticles) as a potential intravenous drug for targeted Am80 delivery to the tumor site. The Am80–PLGA nanoparticles were fabricated using the single-emulsion method in the presence of cationic polyethyleneimine (PEI). The loading efficiency of Am80 in the nanoparticles was controlled by tuning the PEI concentration in the preparation mixture. Nanoparticles with the highest Am80-loading efficiency were dispersible and showed a hydrodynamic diameter of approximately 190 nm in phosphate-buffered saline for up to 2 weeks. The Am80 release from the nanoparticles started in a day and lasted for weeks. The nanoparticles upregulated Meflin expression in human fibroblasts (fHDF/TERT166 cells). These results suggest the potential of Am80–PLGA nanoparticles as a new intravenous anti-tumor drug that can improve the tumor microenvironment, thereby enhancing the efficacy of chemotherapy and immunotherapy.

## 1. Introduction

Tamibarotene (Am80) was developed as a drug with higher specificity to retinoic acid receptors than naturally occurring all-trans retinoic acid, and it was approved in Japan in 2005 for the treatment of acute promyelocytic leukemia [1,2]. Am80 also has potential as an anti-tumor drug in combination with cancer chemotherapeutic and immunotherapeutic drugs because of the following mechanism [3,4,5,6,7,8,9]. First, Am80 upregulates Meflin (glycosylphosphatidyl inositol-anchored protein) expression in cancer-associated fibroblasts (CAFs) [3]. CAF-specific Meflin overexpression improves the tumor microenvironment by transforming cancer-promoting CAFs into cancer-restraining CAFs, which consequently enhance tumor responses to chemotherapy and immunotherapy [3,4,5,6]. Additionally, Am80 exhibits growth-inhibitory effects on certain types of cancer cells [8,9]. Despite such therapeutic efficacy, Am80 is approved only for oral administration because of its poor solubility in water (~7.5 mg/L) and short blood circulation half-life of ~3.3 h; hence, its tumor penetration is limited [10]. For improving the efficacy of Am80, tumor-targeted drug delivery systems (DDSs) for Am80 have been in demand.

Nanoparticles with sizes of 10–300 nm can be carriers in tumor-targeted DDSs because they tend to accumulate at the tumor site after intravenous injection owing to the enhanced permeability and retention (EPR) effect [11,12,13]. Various materials, such as biodegradable polymers, lipids, and inorganic substances (e.g., silica and calcium phosphate), have been used as DDS carriers [14,15,16,17,18]. Among them, poly(lactic-co-glycolic acid) (PLGA)—a copolymer composed of lactic and glycolic acid monomers—has the following advantages as an Am80 carrier for tumor-targeted DDS. First, it exhibits excellent biocompatibility, biodegradability, and bioresorbability and is approved as an injectable product by various national institutions, including the U.S. Food and Drug Administration [19,20,21]. Second, poorly water-soluble drugs can be loaded in PLGA nanoparticles using a simple emulsion method. Additionally, the kinetics of drug release from PLGA nanoparticles can be finely controlled by tuning their physicochemical parameters, such as particle size, drug content, PLGA molecular weight, and lactic-to-glycolic acid ratio [19,20,21].

In this study, we aimed to fabricate PLGA nanoparticles loaded with Am80 (Am80–PLGA nanoparticles) as a promising candidate for a tumor-targeting intravenous drug. Tian et al. previously fabricated PLGA microparticles loaded with Am80 (approximately 7 μm in diameter) using a multistep single-emulsion method and examined their anti-tumor effects against hepatocellular carcinoma via intratumoral injection [22]. Unfortunately, owing to their large particle size (beyond the range of the EPR effect), these microparticles are not suitable for use as tumor-targeted intravenous drugs. For the preparation of smaller PLGA nanoparticles loaded with Am80, we employed a single-emulsion method with a reduced step number and reaction time. However, we faced a hurdle of poor Am80 loading; Am80 was hardly loaded in the PLGA nanoparticles probably due to its anionic nature. To overcome this hurdle, we co-loaded polyethyleneimine (PEI)—a cationic polymer with amine groups—in the nanoparticles, according to the hypothesis that PEI interacts with Am80 carrying carboxyl groups, thereby increasing the Am80-loading efficiency. We made this hypothesis based on the molecular structure of Am80, anionic nature of PLGA, and the known electrostatic attraction between PEI and certain acidic molecules such as DNA, RNA, protein, and indocyanine green [23,24,25,26,27,28,29].

To verify our hypothesis, Am80–PLGA nanoparticles were fabricated using the single-emulsion method in the presence of PEI at five different concentrations. The resulting products were analyzed with regard to shape, size, zeta potential, and Am80-loading efficiency. The selected product was subjected to a preliminary biological assay using human adult skin fibroblasts (fHDF/TERT166 cells)—a cell line of CAF origin—to demonstrate its effect on Meflin expression in cells.

## 2. Materials and Methods

### 2.1. Fabrication of Am80–PLGA Nanoparticles

Am80–PLGA nanoparticles were synthesized via the single-emulsion (antisolvent crystallization) method using dimethyl sulfoxide (DMSO) as a good solvent and water as a poor solvent, as previously described [23]. First, a PLGA solution (100 g/L), an Am80 solution (5 g/L), and a PEI solution (30 g/L) were prepared by dissolving PLGA (Resomer^®^ RG 503H, molecular weight (MW): 24,000–38,000 g/mol, acid-terminated, lactide:glycolide = 50:50 in mol; Merck, Darmstadt, Germany), Am80 (Bio-Techne Corp., Minneapolis, MN, USA), and PEI (MW: 1800, Merck) in DMSO (FUJIFILM Wako Pure Chemical Corporation, Osaka, Japan), respectively. Five DMSO solutions containing PLGA, Am80, and PEI were prepared by mixing DMSO, the 100 g/L PLGA solution, 5 g/L Am80 solution, and 30 g/L PEI solution in various volume ratios (700:200:100:0, 699:200:100:1, 690:200:100:10, 670:200:100:30, and 650:200:100:50). Next, an aqueous solution of 1 wt.% poly(vinyl alcohol) (PVA) was prepared by dissolving PVA (as a stabilizer) (MW: 1500–1800, FUJIFILM Wako Pure Chemical Corporation) in purified water. Preparation mixtures (10 mL) were prepared by dropping the DMSO solution (1 mL) into a 1 wt.% PVA aqueous solution (9 mL) using a syringe (27 G) under stirring. The preparation mixture was stirred (500 rpm) for 15 min at room temperature. After stirring, the generated products were collected via centrifugation (25 °C, 15,000 rpm, 30 min), and washed through decantation of the supernatant, addition of injection water (Fuso Pharmaceutical Industries, Ltd., Osaka, Japan), and redispersion via ultrasonication, which were repeated three times. The resulting products were labeled as **APP0**, **APP1**, **APP10**, **APP30**, and **APP50** according to the volume ratios of the PEI solution (0, 1, 10, 30, and 50, respectively) to the Am80 solution (100) in the DMSO solutions (Table 1). As a control, Am80-free and PEI-free PLGA nanoparticles (represented as PLGANP) were fabricated in the same manner using a DMSO solution prepared by mixing DMSO and the 100 g/L PLGA solution at a volume ratio of 800:200.

### 2.2. Characterization of Products

The product morphologies were examined using a field-emission scanning electron microscope (SU8020; Hitachi High-tech Corporation, Tokyo, Japan). Before the scanning electron microscopy (SEM) analysis, the product dispersions were mounted on a silicon substrate and dried under reduced pressure. The dried products were sputter-coated with carbon. The size (hydrodynamic diameter) distribution and zeta potential of the products were measured using dynamic light scattering (DLS) and electrophoretic light scattering (ELS), respectively. DLS and ELS measurements were performed on the product dispersions using a particle-size analyzer (Zetasizer Nano-ZS, Malvern Instruments Ltd., Worcestershire, UK) after 10 s of ultrasonication.

### 2.3. Determination of Am80-Loading Efficiency

First, the amount of Am80 loaded in the products obtained from a single batch of the preparation mixture (10 mL), referred to as the loaded Am80 amount, was determined as follows. After washing (Section 2.1), the products collected via centrifugation were dissolved in DMSO to prepare test solutions. Using an ultraviolet–visible spectrophotometer (UV-2450; Shimadzu Corporation, Kyoto, Japan), absorbance of the test solutions was measured at 290 nm, which corresponds to the main absorption peak of Am80. In each measurement, a standard curve (see Figure S1a for a typical standard curve) was produced using standard solutions with various Am80 concentrations which were prepared by diluting the 5 g/L Am80 solution (prepared in Section 2.1.) with DMSO. The amount of Am80 in the test solution, i.e., the loaded Am80 amount was determined using an absolute calibration method based on the standard curve.

The Am80-loading efficiency for each product was calculated as a percentage of the loaded Am80 amount among the total amount of Am80 added to the preparation mixture (10 mL). Four independent batches were used to obtain the average and standard deviation (SD).

### 2.4. Am80 Release Assay

The release of Am80 from the selected product (**APP10**) was investigated in phosphate-buffered saline (PBS) at 37 °C using a dialysis membrane method. After washing (Section 2.1), the product dispersion was freeze-dried to obtain a powdered product. The powdery product was resuspended in PBS (FUJIFILM Wako Pure Chemical Corporation, pH 7.1~7.3, KCl: 200 mg/L, NaCl: 8000 mg/L, KH_2_PO_4_: 200 mg/L, Na_2_HPO_4_: 1150 mg/L) such that the concentration of Am80 in the dispersion was 250 µM. The dispersion (1 mL) with a net Am80 amount of 0.25 µmol was added to a dialysis tube (MW cut off 12,000) (BioTech, Taoyuan, Taiwan), which was kept in 10 mL of PBS in a 25 mL tube. The 25 mL tube was incubated with shaking at 80 rpm at 37 °C. After incubation for 0, 1, 3, 6, 24, 72, 120, 168, 336, and 504 h, an aliquot of 1 mL was sampled from 10 mL of PBS (outside the dialysis tube), and fresh PBS (1 mL) was added. The amount of Am80 in the sampled PBS was determined using high-performance liquid chromatography (HPLC) with a Prominence system (Shimadzu Corporation) equipped with an InertSustain AQ-C18 column (GL Sciences Inc., Tokyo, Japan). The mobile phase contained 0.1% phosphoric acid (FUJIFILM Wako Pure Chemical Corporation) and methanol (FUJIFILM Wako Pure Chemical Corporation) (at a volume ratio of 20:80). The flow rate of the mobile phase was 1 mL/min, with a column temperature maintained at 40 °C. Standard solutions with various Am80 concentrations were prepared by diluting the 5 g/L Am80 solution (prepared in Section 2.1) with PBS. A standard curve (Figure S1b) was obtained by measuring the absorbance of these standard solutions at 290 nm. The Am80 in the sampled PBS was quantified by measuring the absorbance at 290 nm using the absolute calibration method based on the standard curve. The quantified Am80 amount was used to calculate the percentage of the transported Am80, i.e., percentage of the amount of Am80 released from the product and diffused into 10 mL of the outside PBS through the dialysis membrane among the total net amount of Am80 added to the dialysis tube. For comparison, the diffusion of free Am80 through the dialysis membrane was investigated using the same procedure except that an Am80 solution diluted by PBS (250 µM, 1 mL) was added to the dialysis tube instead of the product dispersion. Three independent batches were used to obtain the average and SD values.

### 2.5. Particle Stability Test

Particle stability of the selected product (**APP10**) was investigated in PBS. The powdery product (freeze-dried in the same manner as described in Section 2.4) obtained from a single batch of the preparation mixture (10 mL) was resuspended in 10 mL of PBS (same product used in Section 2.4). The product dispersions (1 mL per vial) were incubated with shaking at 80 rpm at 37 °C. After incubation for 0, 24, 72, 168, and 336 h, the product dispersions were ultrasonicated for a few seconds and used for digital camera photography (TOUGH TG-6, Olympus Corporation, Tokyo, Japan) and DLS measurements.

### 2.6. Cell Culture with Products

The fHDF/TERT166 cells (Evercyte, Vienna, Austria) were cultured in Dulbecco’s modified Eagle’s medium (Nacalai Tesque, Kyoto, Japan) supplemented with 10% fetal bovine serum (FBS) (Nichirei Biosciences Inc., Tokyo, Japan) at 37 °C in a humidified incubator with 5% CO_2_. Am80 was dissolved in DMSO to prepare a 10 mM stock solution. After washing (Section 2.1), the selected products (**APP10** and PLGANP) were resuspended in injection water (10 mL). The product dispersions (3 mL per vial) were freeze-dried to obtain powdery products. One day before use, 1 mL of sterile saline was added to each vial, followed by ultrasonication until no visible solids remained. The concentration of Am80 in the **APP10** dispersion was adjusted to 100 μM.

The fHDF/TERT166 cells were seeded in six-well plates and cultured until they reached superconfluency. The culture medium (3 mL per well) was replaced with fresh medium containing one of the following supplements. DMSO group (negative control): DMSO was added to achieve a final concentration of 0.01%, along with 30 μL of sterile saline. Am80 group (positive control): Am80 (from the 10 mM DMSO stock) was added to achieve a final concentration of 1 μM, along with 30 μL of sterile saline. PLGANP group: 30 μL of the PLGANP dispersion was added, along with DMSO (final concentration of 0.01%). **APP10** group: 30 μL of **APP10** dispersion (corresponding to a final Am80 concentration of 1 μM) was added, along with DMSO (final concentration of 0.01%). The DMSO concentration was adjusted to 0.01% in all groups, and the volume of the product dispersion or saline was kept consistent across conditions. After 48 h of culture with supplementation, the cells were harvested for Western blot analysis, as described in Section 2.7.

### 2.7. Western Blot Analysis

Cells were lysed in lysis buffer containing 30 mM Tris-HCl (pH 7.4) (Nacalai Tesque), 120 mM NaCl (Nacalai Tesque), 1 mM ethylenediaminetetraacetic acid (EDTA) (Dojindo Laboratories, Kumamoto, Japan), 1% Triton-X 100 (FUJIFILM Wako Pure Chemical Corporation), 20 mM β-glycerophosphate (Sigma-Aldrich, St. Louis, MO, USA), and 1 mM p-(amidinophenyl) methanesulfonyl fluoride hydrochloride (Sigma-Aldrich) supplemented with Complete Protease Inhibitor (Roche, Basel, Switzerland) and PhosSTOP Phosphatase Inhibitor cocktails (Roche). Lysates were clarified via centrifugation at 12,000 *g* for 10 min at 4 °C, followed by the addition of sodium dodecyl sulfate (SDS) sample buffer (10 mM Tris-HCl (Nacalai Tesque), 2% SDS (FUJIFILM Wako Pure Chemical Corporation), 2 mM EDTA (Dojindo Laboratories), 0.02% bromophenol blue (Nacalai Tesque), 6% glycerol (FUJIFILM Wako Pure Chemical Corporation; pH 6.8) and separation via SDS-polyacrylamide gel electrophoresis. Proteins were transferred onto nitrocellulose membranes, blocked with 5% milk in PBS containing 0.05% Tween 20, incubated with primary antibodies, and detected using horseradish peroxidase-conjugated secondary antibodies (Agilent, Santa Clara, CA, USA). Rabbit polyclonal anti-Meflin antibody (Atlas Antibodies; Cat. No. HPA050811; 1:1000 dilution, Stockholm, Sweden) and mouse monoclonal anti-β-actin antibody (Merck; Cat. No. A5316, 1:2000 dilution) were used as primary antibodies. Western blot signals in the bands of β-actin and Meflin were quantified by densitometry using ImageQuant TL software (Ver. 7.0) (Cytiva, Marlborough, MA, USA).

### 2.8. Statistical Analysis

Statistical analysis was performed using OriginPro software (Ver. 2025) (OriginLab Corporation, Northampton, MA, USA). Differences among groups were analyzed using a one-way ANOVA followed by Fisher’s least significant difference post hoc test. A *p*-value < 0.05 was considered to indicate statistical significance.

## 3. Results and Discussion

### 3.1. Physicochemical Properties of Products

The products were synthesized via the single-emulsion method using the DMSO solutions containing PLGA, Am80, and various concentrations of PEI (Table 1), and 1 wt.% PVA aqueous solution. We varied the PEI concentration in the preparation mixture based on the hypothesis that PEI (a cationic polymer with amine groups) interacts with Am80, which contains carboxyl groups, thereby increasing the Am80-loading efficiency of the nanoparticles through electrostatic attraction.

**Figure 1:**
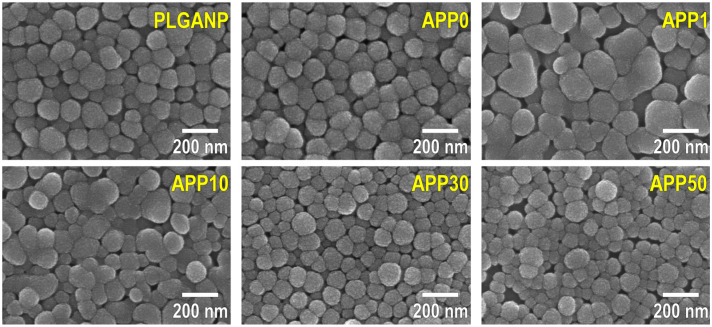
SEM images of PLGANP, **APP0**, **APP1**, **APP10**, **APP30**, and **APP50**.

**Figure 2:**
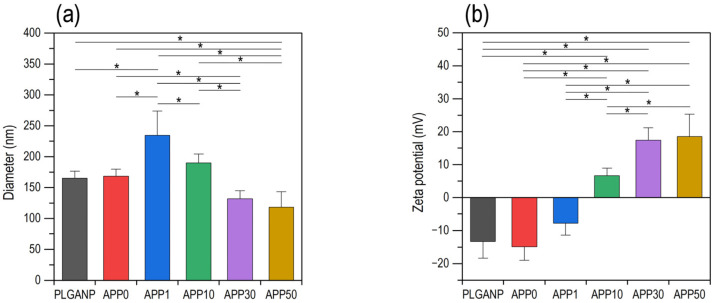
(**a**) Number-average hydrodynamic diameters and (**b**) zeta potentials of PLGANP, **APP0**, **APP1**, **APP10**, **APP30**, and **APP50** in injection water (average + SD (absolute value), *n* = 4, * *p* < 0.05).

The resulting products were water-dispersible PLGA nanoparticles, whose shape, size, and surface charge varied with the PEI concentration in the preparation mixture. SEM images of the products are shown in Figure 1. The primary particle sizes of **APP0**, **APP1**, **APP10**, **APP30**, and **APP50** were in the range of 50–250 nm. **APP0**, **APP30**, and **APP50** were nearly spherical nanoparticles similar to PLGANP, whereas **APP1** and **APP10** were more irregular in shape and larger than the other nanoparticles. According to the DLS measurements, these nanoparticles were dispersed in water, regardless of the PEI concentration in the preparation mixture (Figure S2a). As shown in Figure 2a, the number-average hydrodynamic diameters of PLGANP, **APP0**, **APP1**, **APP10**, **APP30**, and **APP50** were 165 ± 11 nm, 168 ± 12 nm, 234 ± 40 nm, 190 ± 14 nm, 132 ± 13 nm, and 119 ± 24 nm (average ± SD, *n* = 4), respectively, corresponding to the SEM observation results (Figure 1). All nanoparticles exhibited monodispersity in water according to their particle size distributions (Figure S2a) and polydispersity index (PDI) values, i.e., 0.059, 0.047, 0.134, 0.130, 0.055, and 0.096 (average, *n* = 4) for PLGANP, **APP0**, **APP1**, **APP10**, **APP30**, and **APP50**, respectively (PDI < 0.2 indicates monodispersity, from the instrument manual). As shown in Figure 2b and Figure S2b, PLGA nanoparticles (PLGANP) prepared from the preparation mixture without PEI or Am80 exhibited a relatively large negative zeta potential, likely because of the terminal acidic groups of PLGA on their surfaces [30]. The addition of Am80 to the preparation mixture hardly affected the zeta potential of the resulting nanoparticles, considering the similarly negative zeta potentials of PLGANP and **APP0**. The zeta potential of the nanoparticles increased from −14.9 ± 4.1 mV (**APP0**) to 18.5 ± 6.8 mV (**APP50**) with an increase in the PEI concentration in the preparation mixture. This could have been caused by an increase in the amount of positively charged PEI (a cationic polymer with amino groups) co-loaded in the nanoparticles. **APP1** and **APP10** exhibited relatively low absolute zeta potentials below 10 mV. The weaker electrostatic repulsion between these nanoparticles accounts for their morphological features; that is, they are more irregular in shape and larger (Figure 1).

**Figure 3:**
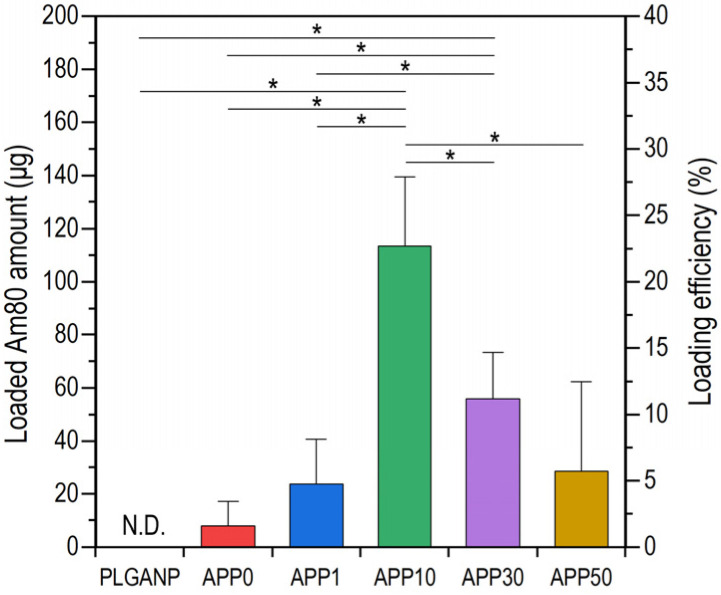
Loaded Am80 amounts (*y*-axis on the left) and Am80-loading efficiencies (*y*-axis on the right) of PLGANP, **APP0**, **APP1**, **APP10**, **APP30**, and **APP50** obtained from a single batch of the preparation mixture (10 mL) (average + SD, *n* = 4, * *p* < 0.05).

The Am80-loading efficiency in the nanoparticles was controlled by tuning the PEI concentration in the preparation mixture. As shown in Figure 3, all nanoparticles except PLGANP contained a certain amount of Am80, indicating that these were Am80–PLGA nanoparticles. The loaded Am80 amount (*y*-axis on the left) and Am80-loading efficiency (*y*-axis on the right) in the nanoparticles increased with the PEI concentration in the preparation mixture up to a certain point (**APP0** < **APP1 < APP10**); however, they decreased at higher PEI concentrations (**APP10** > **APP30** > **APP50**). The increased Am80-loading efficiency in the nanoparticles at lower PEI concentrations was probably due to the increased amount of co-loaded PEI resulting from the electrostatic attraction between the carboxyl groups of Am80 and the amino groups of PEI. The reason for the decreased Am80-loading efficiency at higher PEI concentrations is unclear. Possible reasons include the reduced room for Am80 in PEI-enriched nanoparticles and the inhibition of nanoparticle formation (reduced number of nanoparticles) in the presence of excess PEI.

From the products prepared in this study, we selected **APP10** as Am80–PLGA nanoparticles with the highest Am80-loading efficiency for further investigation.

### 3.2. Particle Stability of Am80–PLGA Nanoparticles

The size of Am80–PLGA nanoparticles (**APP10**) was maintained in PBS for up to 2 weeks. From DLS measurements (Figure S3a) and photographic observations (Figure S3b), **APP10** formed monodisperse suspension even after 336 h (2 weeks) of incubation in PBS at 37 °C. The number-average hydrodynamic diameter of **APP10** was kept unchanged (approximately 190 nm) throughout the stability test. These results indicate that the hydrolytic degradation of the PLGA matrix in **APP10** was so slow that the basic framework of the nanoparticles did not change largely during this stability test. This is consistent with a previous result that PLGA nanoparticles in PBS maintained their structural integrity during the first 20–30 days, followed by aggregation and deformation [31].

### 3.3. Am80 Release from Am80–PLGA Nanoparticles

**Figure 4:**
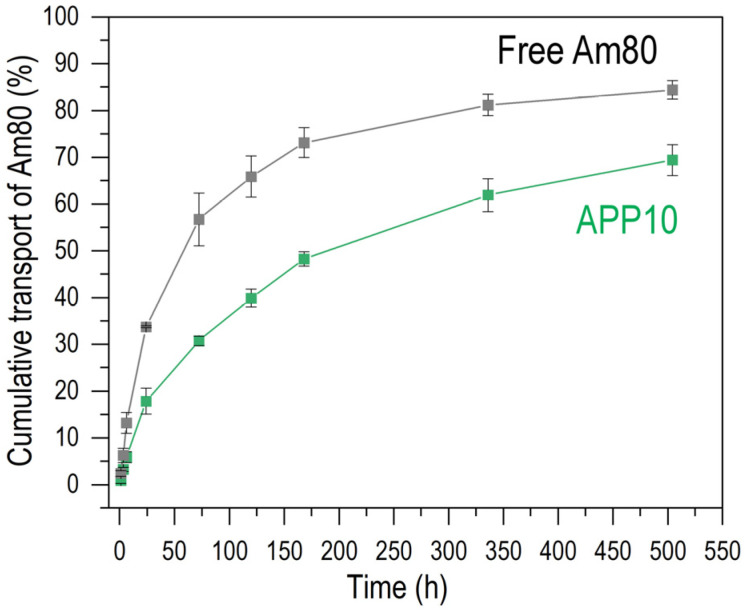
Time course of the percentage of cumulative Am80 transported into the outside PBS from the dialysis tube containing **APP10** (green) and free Am80 (gray), with a net Am80 amount of 0.25 µmol (average ± SD, *n* = 3).

Am80 was released from Am80–PLGA nanoparticles (**APP10**) in PBS. We performed the release assay to confirm whether and how Am80 in the nanoparticle is released in a high ionic strength environment. Figure 4 shows the changes in the percentage of Am80 transported into the outside PBS from the dialysis tube containing **APP10** or free Am80 during 504 h (3 weeks) of incubation. In this assay, the **APP10** dispersion or free Am80 solution (net Am80 amount was 0.25 µmol for both) stored in the dialysis tube was kept in PBS (10 mL). For **APP10**, the concentration of Am80 in the outside PBS increased monotonically over time (Figure 4; green line). The Am80 detected in the outside PBS was released from **APP10** and diffused across the dialysis membrane because nanoparticles larger than 10 nm could not pass through the dialysis membrane (pore size of approximately 2.5 nm). The result indicates that the release of Am80 from the nanoparticles started in a day (at least 17% was released in 24 h) and lasted for weeks. Free Am80 (gray line) exhibited a faster increase in Am80 concentration in the outside PBS than **APP10** (Figure 4). This profile reflected solely the diffusion of free Am80 through the dialysis membrane. Therefore, the slower increase in the Am80 concentration for **APP10** was attributed to the capacity of the nanoparticles to retain Am80 and the process of their Am80 release. The release of Am80 from the nanoparticles should be mediated by desorption from the particulate surface, degradation of the PLGA matrix, and leaking from the PLGA matrix. Considering the result of particle stability test (Figure S3a), effect of the matrix (PLGA) degradation would be relatively small in this assay.

### 3.4. Effect of Am80–PLGA Nanoparticles on Meflin Expression in Human Fibroblasts

**Figure 5:**
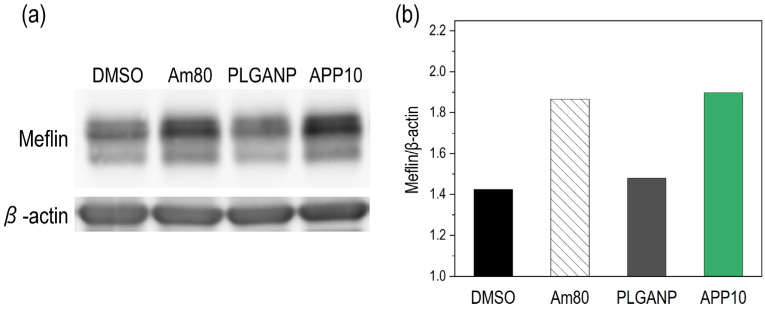
Meflin expression in fHDF/TERT166 cells analyzed via Western blotting. (**a**) Images of Western blot bands of Meflin and β-actin and (**b**) relative expression of Meflin to β-actin (quantified via densitometry) in the cells cultured with DMSO (negative control), Am80 (positive control), PLGANP, and **APP10**.

The Am80–PLGA nanoparticles (**APP10**) upregulated Meflin expression in human fibroblasts. Figure 5 shows the qualitative and quantitative analytical results of Meflin expression in fHDF/TERT166 cells cultured with DMSO (negative control), Am80 (positive control), PLGANP, or **APP10**. The Meflin expression level of PLGANP was similar to that of DMSO (negative control), indicating that PLGA nanoparticles without Am80 had little effect on Meflin expression in cells. In contrast, **APP10** induced a higher level of Meflin expression in the cells than DMSO and achieved a similar Meflin expression level to Am80 (positive control). These results suggest that Am80 was released from the Am80–PLGA nanoparticles under the tested cell culture condition, and the released Am80 maintained its biological activity to enhance Meflin expression.

### 3.5. Future Perspectives and Challenges

As described above, Am80–PLGA nanoparticles were fabricated using the single-emulsion method in the presence of PEI. We revealed that the PEI concentration in the preparation mixture was a controlling factor affecting the size, zeta potential, and Am80-loading efficiency of the resulting Am80–PLGA nanoparticles. Such a significant effect of PEI should be caused by electrostatic interactions between amine groups in PEI and carboxyl groups in Am80. By adjusting the PEI concentration in the preparation mixture, we increased the Am80-loading efficiency (Figure 3), and successfully fabricated Am80–PLGA nanoparticles (**APP10**) that meet some essential requirements for application as a tumor-targeting intravenous Am80-carrier, i.e., size (Figure 1, Figure 2a and Figure S2), water-dispersibility (Figure S2), size stability (Figure S3), and capability to release biologically active Am80 (Figure 4 and Figure 5), as detailed below.

The Am80–PLGA nanoparticles fabricated in this study have potential as intravenous anti-tumor drugs for use in combination with chemotherapeutic and immunotherapeutic drugs because of their properties and anticipated functions. The nanoparticles (**APP10**) with the highest Am80-loading efficiency exhibited water dispersibility and a hydrodynamic diameter of approximately 190 nm (Figure 2a). Water-dispersible nanoparticles of this size can be administered intravenously. Intravenously administrated Am80–PLGA nanoparticles are expected to accumulate at tumor sites while circulating in the blood because of the EPR effect [11,12,13]. The nanoparticles remained similar in size for 2 weeks in PBS (Figure S3), suggesting that they are sufficiently stable to exert the EPR effect. The nanoparticles delivered to the tumor sites should release Am80 for weeks mainly via desorption from the particulate surface and/or leaking from the PLGA matrix. According to the results obtained, the Am80 release from the nanoparticles started in a day and lasted for weeks in PBS (Figure 4), and the released Am80 retained its biological activity (Figure 5). Thus, the released Am80 is considered to induce Meflin upregulation in the surrounding CAFs, which leads to their transformation into cancer-restraining CAFs. An improved tumor microenvironment should enhance the therapeutic efficacy of subsequent chemotherapy and immunotherapy.

The Am80–PLGA nanoparticles would be a solution to address the clinical issues associated with the limited penetration of Am80 into tumor sites. They should allow tumor-targeted delivery of Am80 via intravenous administration, followed by Am80 release at the tumor site. With such a DDS, biologically active Am80 can penetrate tumors, even in deep tissues of the human body, without invasive treatments. It has been reported that CAFs are closely involved in the progression of intractable pancreatic cancer [32,33,34]. Our Am80–PLGA nanoparticles offer a new treatment approach for intractable cancers located deep within the abdomen.

This study has several limitations. First, the composite nanostructure of Am80–PLGA nanoparticles and their Am80 release mechanism are not fully elucidated. Second, this in vitro study is preliminary and insufficient to understand the cell–particle interactions. Third, in vivo behaviors of the nanoparticles, including their blood circulation, tumor accumulation and penetration, and final fate, are yet to be clarified. In addition, therapeutic effects of the nanoparticles and their advantage over free Am80 still remain unclear. In-depth analyses of the nanoparticles, more detailed in vitro experiments, and in vivo experiments using cancer models are needed to clarify the clinical potential of Am80–PLGA nanoparticles.

## 4. Conclusions

Water-dispersible Am80–PLGA nanoparticles were fabricated using a single-emulsion method in the presence of PEI. Their size, zeta potential, and Am80-loading efficiency were controlled by varying the PEI concentration in the preparation mixture. The nanoparticles with the highest Am80-loading efficiency (**APP10**) had a number-average hydrodynamic diameter of approximately 190 nm, which is suitable for intravenous injection and capable of exploiting the EPR effect. The nanoparticles released Am80 in PBS for weeks and upregulated Meflin expression in human fibroblasts. These results demonstrate the potential of Am80–PLGA nanoparticles as tumor-targeted intravenous drugs that can affect CAFs and improve the tumor microenvironment, thereby enhancing the efficacy of chemotherapeutic and immunotherapeutic drugs.

## Figures and Tables

**Table 1:** The volume ratios (parts per thousand by volume) of DMSO, PLGA solution, Am80 solution, and PEI solution for preparing the DMSO solutions to produce PLGANP, **APP0**, **APP1**, **APP10**, **APP30**, and **APP50**.

Product Name	Liquid Source Ratio of the DMSO Solution			
	DMSO	100 g/L PLGA Solution	5 g/L Am80 Solution	30 g/L PEI Solution
PLGANP	800	200	-	-
**APP0**	700	200	100	-
**APP1**	699	200	100	1
**APP10**	690	200	100	10
**APP30**	670	200	100	30
**APP50**	650	200	100	50

## Data Availability

The original contributions presented in the study are included in the article, further inquiries can be directed to the corresponding authors.

## Supplementary Materials

The following supporting information can be downloaded at: https://www.mdpi.com/article/10.3390/jfb16100368/s1, Figure S1: Standard curves prepared in (a) ultraviolet–visible spectrophotometry and (b) HPLC for quantifying Am80 in the test solutions; Figure S2: (a) Size distributions (by DLS) and (b) zeta potential distributions (by ELS) of PLGANP, **APP0**, **APP1**, **APP10**, **APP30**, and **APP50**; Figure S3: (a) Size distributions (by DLS) and (b) digital photographs of APP10 dispersed in PBS after incubation for 0, 24, 72, 168, and 336 h at 37 °C.
