# Supplementary material for: Polyethyleneimine-Assisted Fabrication of Poly(Lactic-Co-Glycolic Acid) Nanoparticles Loaded with Tamibarotene (Am80) for Meflin Expression Upregulation
Source: J Funct Biomater. 2025 Oct 1;16(10):368. doi: 10.3390/jfb16100368 (PMC12565359; doi:10.3390/jfb16100368)
Supplement: Supplementary file 1 [file jfb-16-00368-s001.zip › jfb-3809174-supplementary.pdf]

Supplementary Material

Polyethyleneimine-Assisted Fabrication of Poly(Lactic-Co-Glycolic Acid) Nanoparticles Loaded with Tamibarotene (Am80) for Meflin Expression Upregulation

Tomoya Inose[1,*], Tadashi Iida[2,3], Hiroki Kawashima[2], Atsushi Enomoto[3], Maki Nakamura[1,*] and Ayako Oyane[1]

[1] *Research Institute of Core Technology for Materials Innovation, National Institute of Advanced Industrial Science and Technology (AIST), AIST Tsukuba Central 5, 1-1-1 Higashi, Tsukuba 305-8565, Japan*

[2] *Department of Gastroenterology and Hepatology, Graduate School of Medicine, Nagoya University, 65 Tsurumai-cho, Shouwa-ku, Nagoya 466-8550, Japan*

[3] *Department of Pathology, Graduate School of Medicine, Nagoya University, 65 Tsurumai-cho, Shouwa-ku, Nagoya 466-8550, Japan*

* Correspondence: t.inose@aist.go.jp (T.I.); ma-ki-nakamura@aist.go.jp (M.N.)

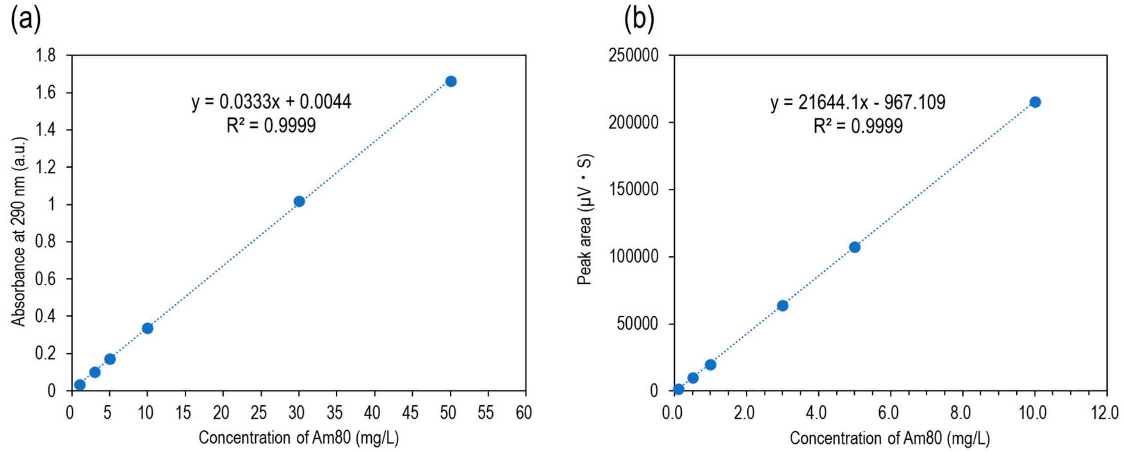

Figure S1. Standard curves prepared in (a) ultraviolet–visible spectrophotometry and (b) HPLC for quantifying Am80 in the test solutions.

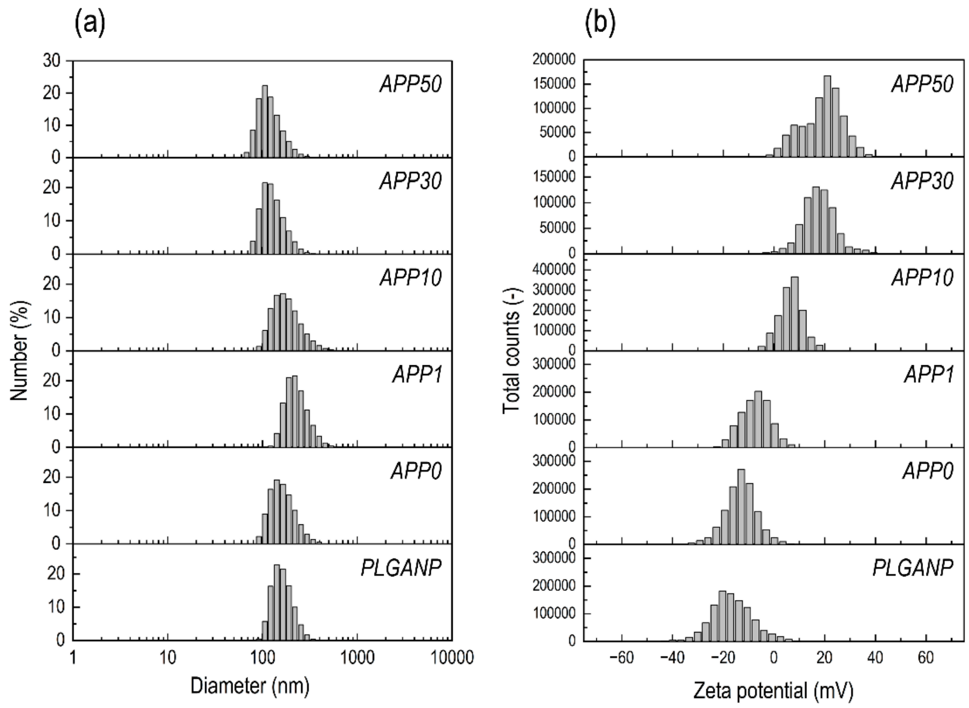

Figure S2. (a) Size distributions (by DLS) and (b) zeta potential distributions (by ELS) of PLGANP, **APP0**, **APP1**, **APP10**, **APP30**, and **APP50**.

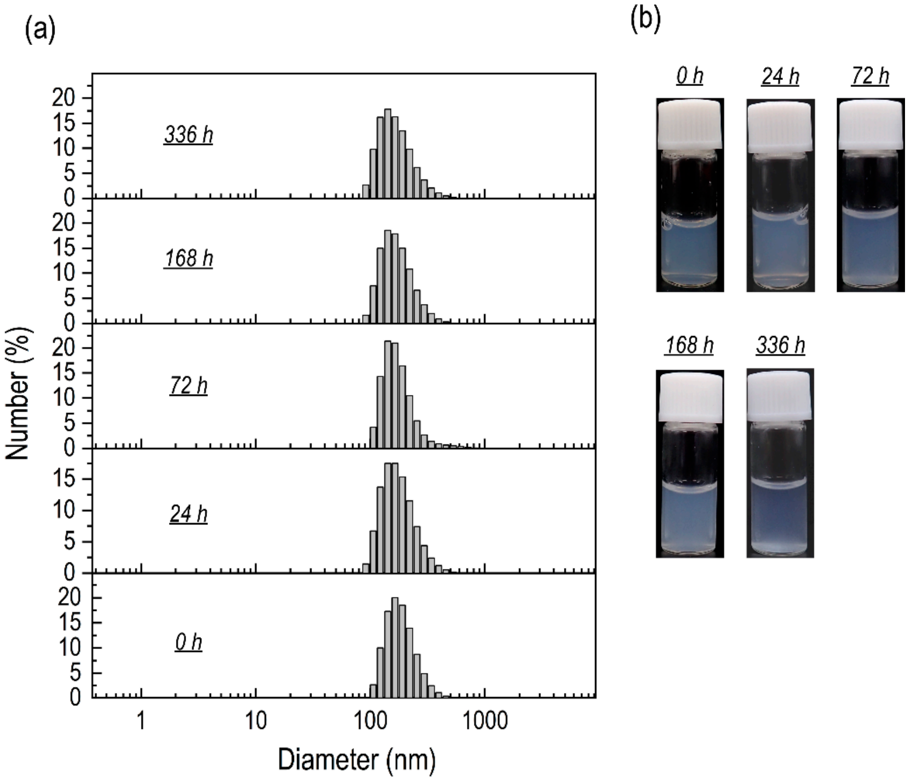

Figure S3. (a) Size distributions (by DLS) and (b) digital photographs of **APP10** dispersed in PBS after incubation for 0, 24, 72, 168, and 336 h at 37°C.
